# Real-Time and Online Lubricating Oil Condition Monitoring Enabled by Triboelectric Nanogenerator

**DOI:** 10.1021/acsnano.1c02980

**Published:** 2021-06-25

**Authors:** Jun Zhao, Di Wang, Fan Zhang, Yuan Liu, Baodong Chen, Zhong Lin Wang, Jinshan Pan, Roland Larsson, Yijun Shi

**Affiliations:** † Division of Machine Elements, Luleå University of Technology, Luleå, SE-971 87 Sweden; ‡ College of Mechanical and Electrical Engineering, Beijing University of Chemical Technology, Beijing 100029, P. R. China; § Department of Engineering and Design, School of Engineering and Information, University of Sussex, Brighton, BN1 9RH, United Kingdom; ∥ CAS Center for Excellence in Nanoscience, Beijing Key Laboratory of Micro-Nano Energy and Sensor, Beijing Institute of Nanoenergy and Nanosystems, Chinese Academy of Sciences, Beijing, 101400, P. R. China; ⊥ Division of Surface and Corrosion Science, Department of Chemistry, KTH Royal Institute of Technology, Stockholm, SE-100 44, Sweden

**Keywords:** lubricating oils, condition monitoring, triboelectric nanogenerator, TENG, smart machines

## Abstract

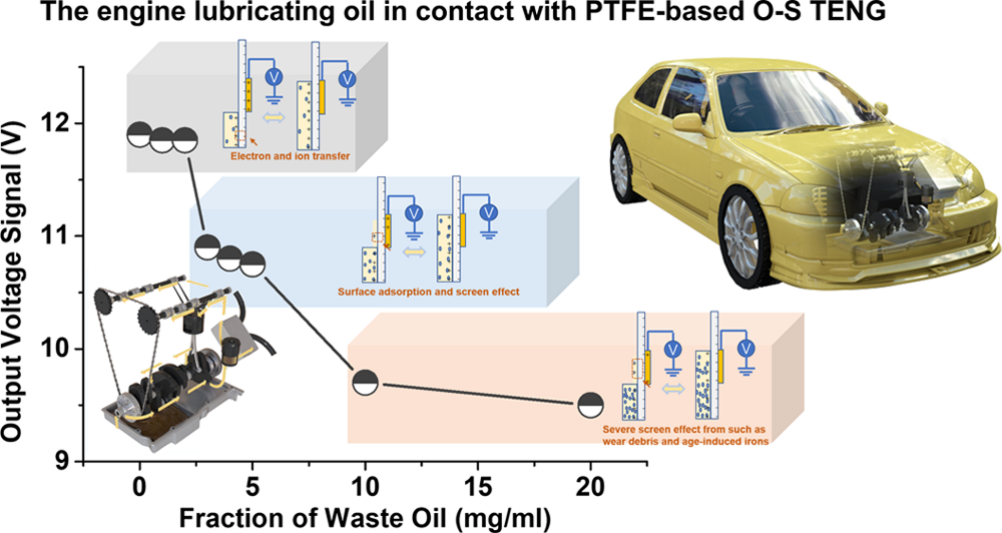

An intelligent monitoring lubricant is essential for the development of smart machines because unexpected and fatal failures of critical dynamic components in the machines happen every day, threatening the life and health of humans. Inspired by the triboelectric nanogenerators (TENGs) work on water, we present a feasible way to prepare a self-powered triboelectric sensor for real-time monitoring of lubricating oils *via* the contact electrification process of oil–solid contact (O–S TENG). Typical intruding contaminants in pure base oils can be successfully monitored. The O–S TENG has very good sensitivity, which even can respectively detect at least 1 mg mL^–1^ debris and 0.01 wt % water contaminants. Furthermore, the real-time monitoring of formulated engine lubricating oil in a real engine oil tank is achieved. Our results show that electron transfer is possible from an oil to solid surface during contact electrification. The electrical output characteristic depends on the screen effect from such as wear debris, deposited carbons, and age-induced organic molecules in oils. Previous work only qualitatively identified that the output ability of liquid can be improved by leaving less liquid adsorbed on the TENG surface, but the adsorption mass and adsorption speed of liquid and its consequences for the output performance were not studied. We quantitatively study the internal relationship between output ability and adsorbing behavior of lubricating oils by quartz crystal microbalance with dissipation (QCM-D) for liquid–solid contact interfaces. This study provides a real-time, online, self-powered strategy for intelligent diagnosis of lubricating oils.

Human civilization and cultural communication benefit greatly from the development of modern industry,^1^ in which automobiles, trains, vessels, and aircrafts play an important role in terms of convenience, efficiency, and safety.^2−4^ Unexpected and fatal failures of critical dynamic components in machines happen every day, threatening the life and health of humans. Hence, reliable condition monitoring (CoMo) will thus be of importance in order to make sure that the machine services can be reliably delivered.^4−8^ With the industry 4.0 automation increase, there is a need for smart machines able to understand or sense the failures and make decisions accordingly by artificial intelligence and machine learning.^1,2,9^

Lubricants can extend machine lifetimes by orders of magnitude, which is of great significance for energy conservation and emission reduction.^10−14^ Using lubricants is the most effective way to control friction and wear, because moving mechanical interfaces are commonly lubricated and separated by fluid lubricating films. Therefore, the lubricant is an important source of information in the strategy to detect machine failures, comparable to the role of human blood in the detection and prevention of diseases.^15,16^ The real-time detection of lubricants can eliminate the need of costly machine shutdowns for inspection, which would otherwise be required to avoid the possibility of catastrophic component failure during operation.

Intruding contaminants from thermal oxidation, wear debris, carbon deposition, fuel, and moisture often exist in lubricating oils and are mainly issues causing lubrication failure. For example, the heat produced in the engine segment should influence the oil performance. Low oxidation stability of lubricating oils may result in oil acidification and carbon deposition under high-temperature aging.^17−19^ Under operation, the fraction of wear debris in oils gradually increases when frictional surfaces are worn (Fe and Cu debris),^10^ and the size of the debris is in the range of 10–100 μm.^20^ Fuel or water may permeate engine lubricating oils *via* the frictional interface of piston/cylinder, when the piston is reciprocating inside the cylinder.^21−23^ Fuel and water not only damage the oil quality and lubrication performance, but also corrode the machine.

There are many methods used as monitoring sensors for the quality of lubricating oils. Some examples are optical methods, acoustic emission detection methods and electromagnetic-inducted technologies.^15,20,24^ Traditionally, some contaminant ingressions can always be reflected by a change of dielectric constant of lubricant, therefore the contaminants can be detected timely by monitoring the dielectric constant of the used lubricant. However, these methods can only provide limited information on the progression of ferrous wear debris by off-line monitoring and with relatively low accuracy. The present sensors can only detect large particles with a diameter of 100∼300 μm at the lowest concentration of about 1 mg mL^–1^, and can only detect the water contaminant down to 0.33 wt %.^15,20^ Most conventional detection sensors are quite large and unwieldy, and need installation or attachment to equipment systems, potentially causing interference with monitoring systems. Due to reliance on external power sources, the energy consumption is a challenge for their miniaturization and weight reduction, and they have limited-service life. It is much desired to develop a self-powered, high-sensitivity, small, and even flexible detection system for real-time, online monitoring of lubricating oils.

The triboelectric nanogenerator (TENG), based on the conjunction of triboelectrification and electrostatic effects, was developed for energy harvesting and self-powered monitoring by Wang and co-workers in 2012.^25^ TENG-based sensors have successfully been used as mechanical sensors for detecting water wave,^26,27^ liquid flow rate,^28^ and organic^29^ and ion concentration^30^ based on liquid–solid contact electrification. Inspired by the works on water-based systems, we propose a method to develop TENG for oil condition monitoring. The electrification process of liquid–solid contact can produce a surface charge on a large scale *via* electron and ion transfer on liquid–solid contact interfaces.^8,31−37^ It has been found that electron transfer is the dominating mechanism for the triboelectrification process in solid–solid cases,^38^ so lubricating oils with no ions can generate a certain amount of charges by electron transfer between oil–solid interfaces.^39,40^

In this study, we present a feasible way to prepare and apply a self-powered triboelectric sensor using oil–solid interacting TENG (O–S TENG) for real-time, online monitoring of lubricating oils. First, three kinds of pure base oils (polyalphaolefin 6 (PAO-6), paraffin and rapeseed oils) and typical contaminant ingressions (i.e., thermal aging, wear debris, carbon deposition, diesel oil, and water) are used. *Via* the triboelectrification on liquid–solid interface, the electric signals generated from the contact tribo-layers can detect lubricating oil conditions. The working mechanism of the O–S TENG is illustrated. On the basis of the model study, a sensor is developed to be used as real-time and online monitoring of an engine lubricating oil in actual operation. Results show that this sensor has great potential in building a self-powered, real-time, and online monitoring system for lubricating oils.

## Results and Discussion

### Structure and Working Principle of the O–S TENG

**Figure 1:**
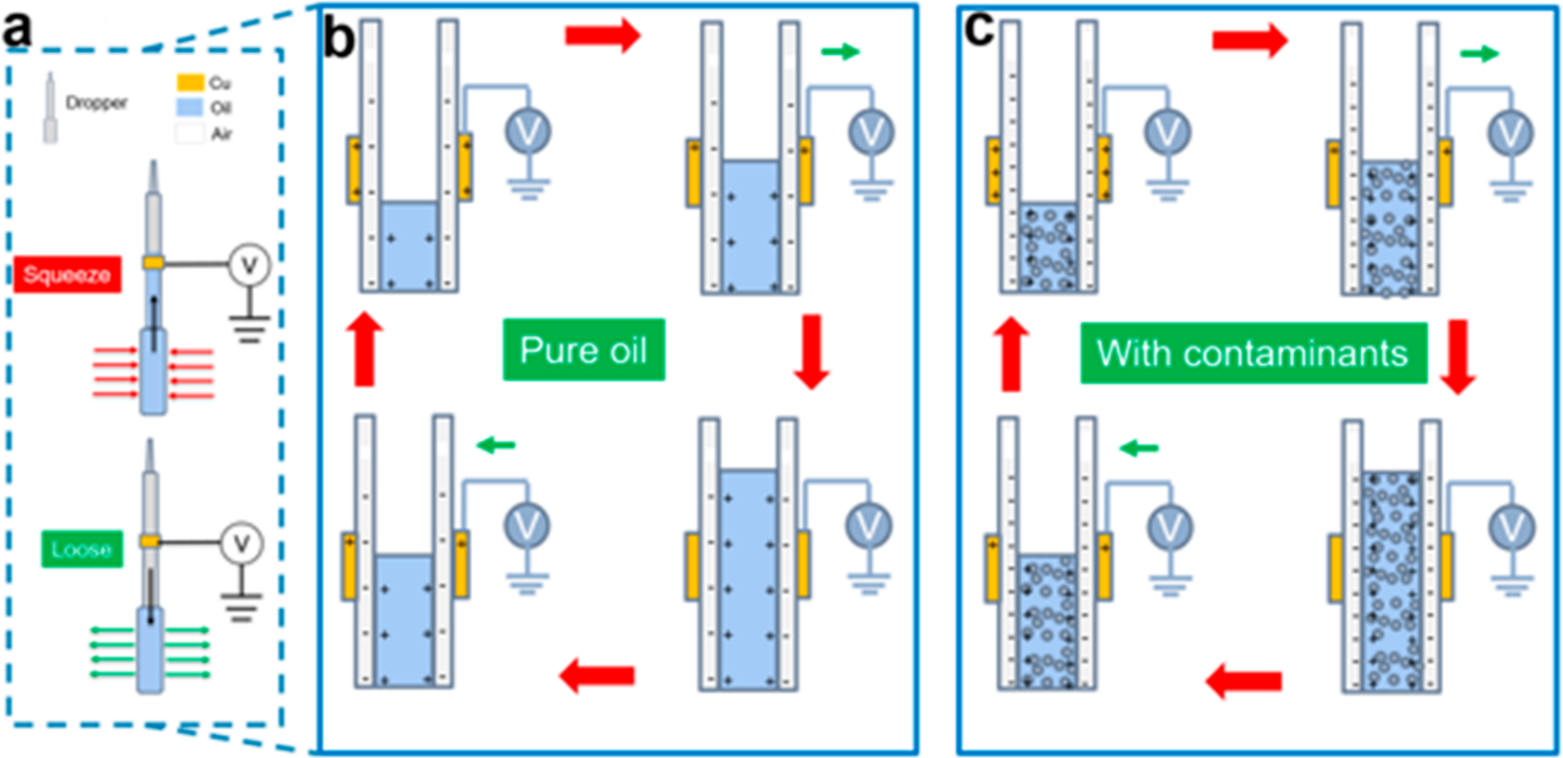
Structure illustration and working principle of the O–S TENG. (a) Structural schematic diagram of the developed O–S TENG sensor. (b) Typical output signal generated by the interfacial interaction between a pure lubricating oil and a Cu electrode. (c) Output generation of the lubricating oil with contaminant ingressions.

The triboelectric sensor for detecting contaminants in oils is developed by using a dropper covered with a copper foil as shown in Figure S1. Typically, polytetrafluoroethylene (PTFE), low-density polyethylene (LDPE), or glass (GLASS) tubes are used as substrates, and the copper layer is deposited on the outside surface of the tubes, forming the single electrode TENG sensor. In the case of liquid flow sensor in Figure 1a, oil flows (PAO-6, paraffin, or rapeseed oils) are squeezed or loosed from the grip tips of droppers, then the flow proactively passes through the TENG surface, and the oil flow motion will generate electric output signals. As shown in Figure 1b, the output signal generation from the developed O–S TENG is based on both triboelectrification and electrostatic induction.^41,42^ Because of the interaction with Cu, the nonmetal surface layers of PTFE and LDPE retain a layer of negative bound charges, while GLASS surface keeps positive charges as shown in Supporting Information, Figure S2 due to their different positions in triboelectric series.^43,44^ The initial voltage outputs for PTFE and LDPE-based TENGs are mainly positive after a holding stage with weak signal drift. Conversely, the GLASS-based TENG displays a relative negative voltage signal shown in Figure S3. The key to monitor the oil condition is from the amplitude of output values and the variation tendency of voltage signal in this study, thus, the typical voltage outputs are uniformly processed in one baseline (zero axis).

When the oil molecules initially approach a virgin surface that has no pre-existing surface charges, initial electron transfer occurs to make the solid surface be charged (Figure S4). The nonmetal surface will attract the charged oil molecules to form an electric double layer (EDL) that will screen the electrostatic inducted charges of the nonmetal layers. Therefore, electrons will flow from the ground to the Cu electrode under short-circuit condition for an electric equilibrium. When the flow leaves the nonmetal layer, the screen effect will disappear and electrons will flow from Cu to the ground to reach a new electric equilibrium. As illustrated in Figure 1c, the contaminants in a lubricating oil will change the electrification process performance of the oil, and can be reflected from the O–S TENG electric output. On the basis of the above working mechanism, O–S TENG has the potential to monitor the condition of lubricating oils. To clarify the role of contaminants in altering the output signal of O–S TENG, the influence factors, that is, thermal aging, wear debris, deposited carbons, fuel oil, and water are systematically studied as summarized in Table S1.

### Base Lubricating Oil Condition Monitoring by the O–S TENG

**Figure 2:**
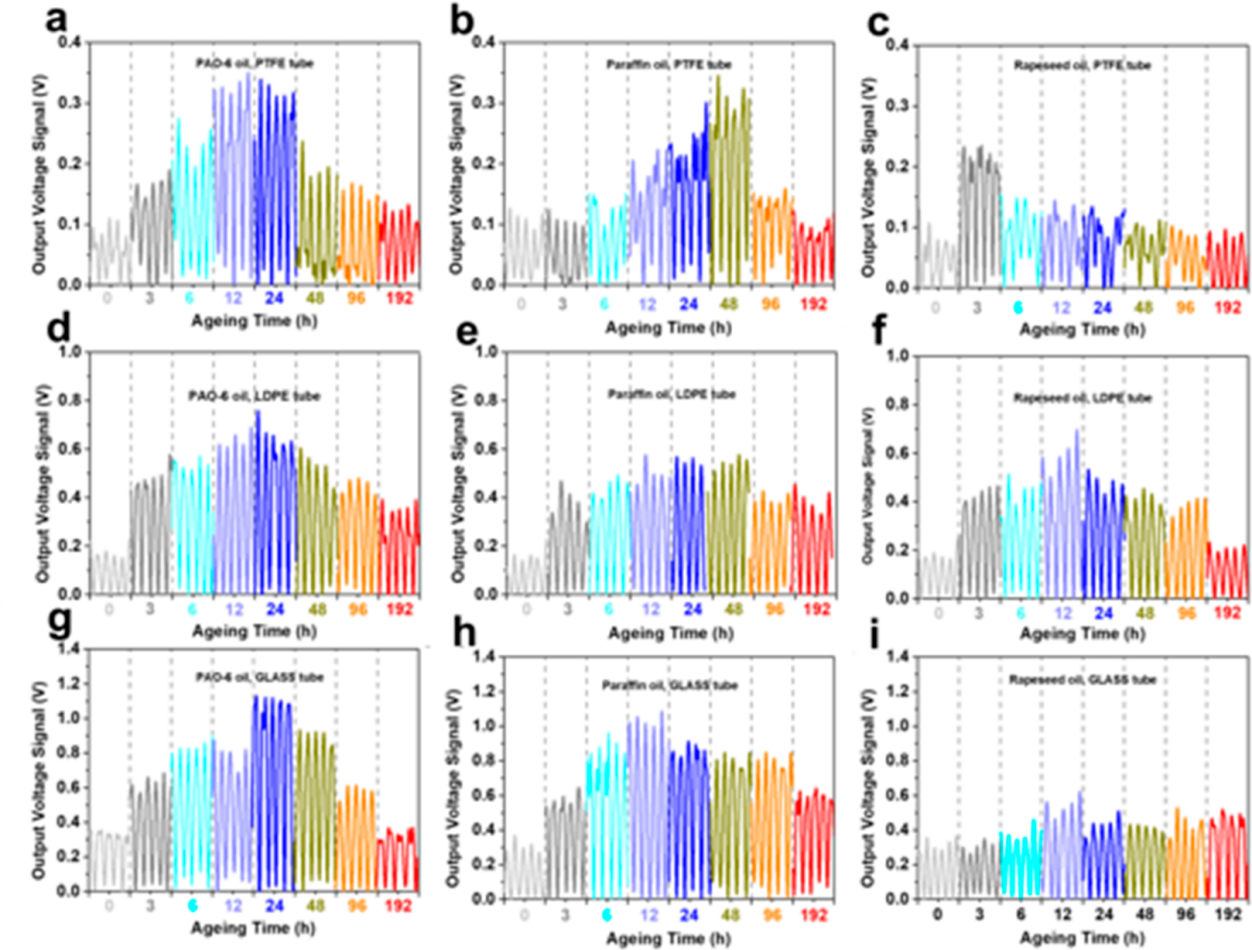
Typical signal curves in 5 s for thermal aging affecting on the output voltages of O–S TENG monitoring pure base oils. PTFE-based O–S TENG monitoring PAO-6 oil (a), paraffin oil (b), and rapeseed oil (c). LDPE-based O–S TENG monitoring PAO-6 oil (d), paraffin oil (e), and rapeseed oil (f); GLASS-based O–S TENG monitoring PAO-6 oil (g), paraffin oil, (h) and rapeseed oil (i).

During operation of the actual equipment, lubricating oils inevitably suffer from thermal oxidation. Thermal aging is therefore an important sign of oil deterioration.^17^ To demonstrate the applicability of the O–S TENG, pure base oils with different aged degrees were first prepared. During the test, the volume of each oil flow in tubes was about 2 mL, and the frequency of the oil flowing through the electrode surface was set at around 1 ± 0.1 Hz by manually squeezing and loosing. Figure 2 shows the voltage outputs of O–S TENG driven by pure base oils aged with various aging time periods (0∼192 h). As shown in Figure 2a–c, the output values of the three pure base oils are about 0.1 V when the oil flows in contact with the PTFE tube. For aged PAO-6 oil flow, the output voltage increases from 0.1 V to 0.33 V with the increase of aging time (up to 12 h). The maximum output value of paraffin oil is about 0.32 V when the aging time is 48 h, whereas the output value of rapeseed oil does not increase after aging for 3 h. As shown in Figure 2d–i, the variation tendency of voltage signals for LDPE and GLASS tubes resemble that for PTFE tubes, which further confirms that thermal aging greatly affects the output of O–S TENG, and the O–S TENG can effectively monitor the aging degree of lubricating oils.

**Figure 3:**
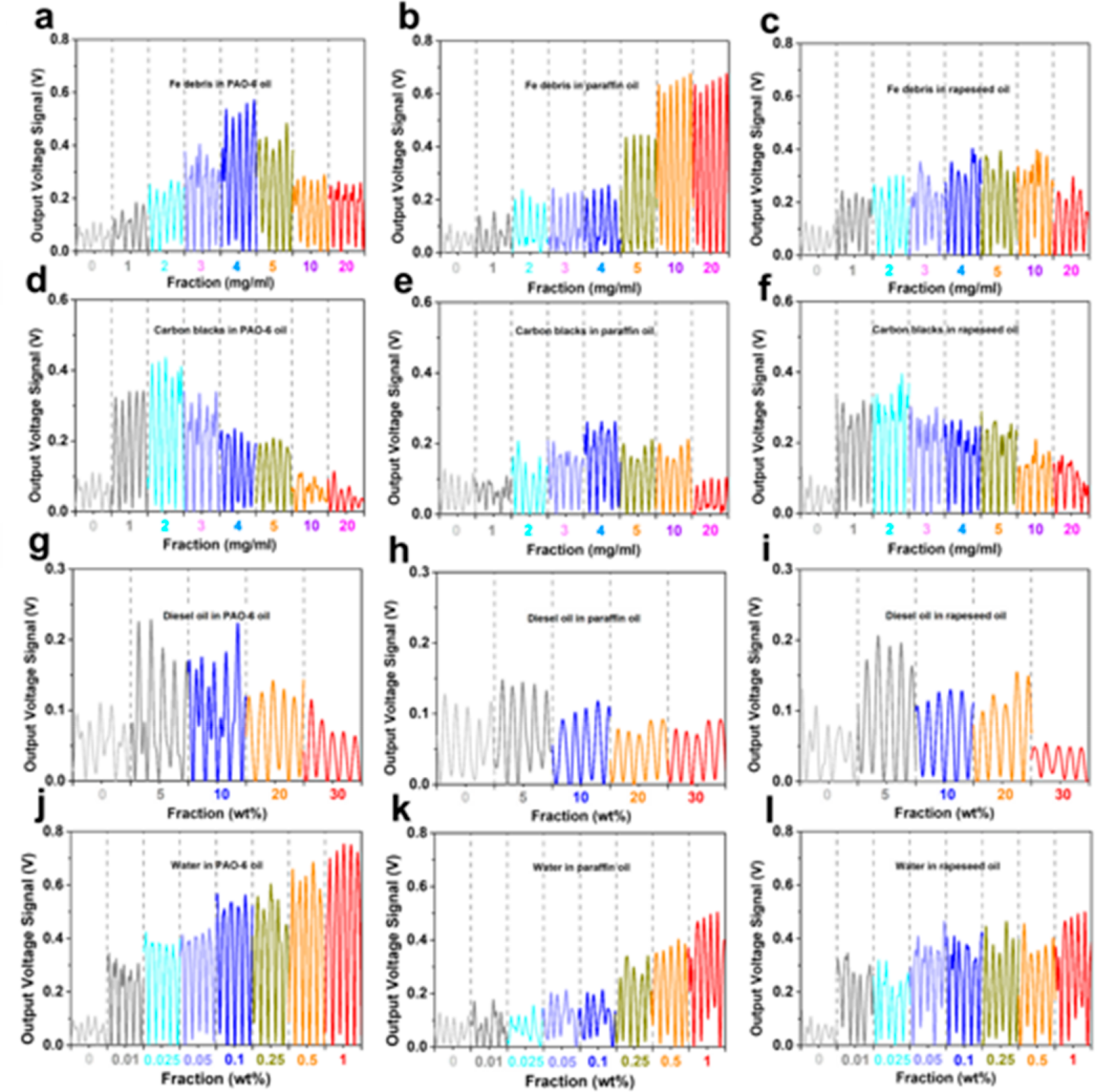
Typical signal curves in 5 s for the output voltage of PTFE-based O–S TENG monitoring pure base oils. Various fractions of Fe debris (0∼20 mg mL^–1^) affecting the O–S TENG output of PAO-6 oil (a), paraffin oil (b), and rapeseed oil (c). Various fractions of carbon blacks (0∼20 mg mL^–1^) affecting the O–S TENG output of PAO-6 oil (d), paraffin oil (e), and rapeseed oil (f). Various fractions of diesel oils (0∼30 wt %) affecting the O–S TENG output of PAO-6 oil (g), paraffin oil (h), and rapeseed oil (i). Various fractions of water (0∼1 wt %) affecting the O–S TENG output of PAO-6 oil (j), paraffin oil (k), and rapeseed oil (l).

Typical contaminant fractions as byproducts of wear process and carbon deposition^15,20^ increase gradually with time causing machine deterioration and even failure. It is critical to monitor the contaminants to avoid catastrophic failure. We dispersed Fe/Cu particles as the wear debris and carbon blacks in base oils to simulate lubricants that undergo the actual wear and carbon deposition processes. The tested fractions and morphologies of Fe/Cu debris and carbon blacks are respectively summarized in Table S1 and Figure S5. As shown in Figure S6, the output values initially increase and then decrease with the increase of fraction, and the variation tendency of voltage output for PTFE-based O–S TENG is in good agreement with those for both LDPE-based and GLASS-based O–S TENGs. Figure 3 panels a–c show the voltage outputs of PTFE-based O–S TENG driven by debris-laden flows with the fraction range from 0 mg mL^–1^ to 20 mg mL^–1^. In contact with Fe debris-laden PAO-6 flow, the O–S TENG device has a maximum output voltage of 0.58 V at a debris fraction of 4 mg mL^–1^. The maximum output voltages are 0.65 V (at 10 mg mL^–1^) and 0.37 V (at 4 mg mL^–1^), respectively for paraffin and rapeseed flows. The voltage value of Cu debris-laden flow also increases with the increase of Cu debris fraction, and then decreases at high fraction in Figure S7. When in contact with carbon black-laden flows shown in Figure 3d–f, the output voltages of base oils also have high peaks with the increase of the fraction, but the critical fraction value is much lower than that of Fe and Cu debris-laden flows. During actual equipment operation, fuel oil in an engine combustor always enters into the engine lubricating oil from the frictional contact area between the cylinder-wall and piston.^45^

The influence of fuel oil (diesel oil) is also studied (Figure 3g–i and Figure S8). It is clearly seen that the voltage value gradually decreases at a high fraction of diesel oil. Oil/water monitoring and separating is a worldwide concerned challenge because of increasing industrial oily wastewater, as well as correlatively frequent accidents.^46−48^ The monitoring of water in oil is an interfacial challenge, and using TENG sensors is an effective way to address this challenge. As shown in Figure 3j–l and Figure S9, the voltage output increases persistently with the fraction of water increasing for all base oils, respectively, passing PTFE-based, LDPE-based, and GLASS-based O–S TEGNs. Especially, for the water-laden flows of PAO-6 and rapeseed oils, the output voltage obviously increases from 0.1 to 0.3 V when the water fraction increases only to 0.01 wt % as shown in Figure 3j and Figure 3l, which means this developed O–S TENG shows a high sensitivity for monitoring water in oils in spite of a very low water fraction.

### Electrical Output Mechanism of Lubricating Oils

According to the output voltage of O–S TENG above, it can be found that the variation tendency of water-laden oil flow is different from those of Fe, Cu debris, and carbon black-laden flows. When the water in the oil flow contacts the O–S TENG surface, the output voltage will increase because the output of water/solid electrification is higher than that of oil/solid electrification as reported in the previous work.^31^ In addition, as is known, the surface tension of water (62∼72 mJ m^–2^) is much higher than that of oil (31∼35 mJ m^–2^),^21,49^ which means the interfacial wettability of the oil flow in contact with the TENG surface is weaker when water is added to the oil, that is, water has greater tendency to leave the surface, which lessens water residues. On the contrary, it can be understood that the output of diesel oil-laden flow obviously decreases at a high fraction, because the diesel oil with very low surface tension (28 mJ m^–2^) is prone to spreading and adsorbing on the TENG surface.^21^ The output performances of aged base oils (aged PAO-6 oil, aged paraffin oil, and aged rapeseed oil) are in good agreement with Fe/Cu debris-laden and carbon black-laden oils, i.e., the output voltage slightly increases and then decreases gradually with the increase of contaminant fraction. With the increase of thermal aging, the color of the oils gradually changes from colorless to orange for both PAO-6 and paraffin oils shown in Figure S10. It means these two pure oils have been severely oxidized after long aging time and they are less stable than rapeseed oil. According to the results of Fourier transform infrared spectroscopy (FTIR) in Figure S11, there are many oxygen-containing components (carboxylate, carbonyl (1158 cm^–1^), and hydroxy (3470 cm^–1^) groups) especially of carboxylates generated in PAO-6 and paraffin oils after the long aging time (48∼192 h).^50,51^ Therefore, these aged oils display much higher polarity with a larger concentration of ions than pure base oils, so the positive relation between the output value and the thermal aging time is due to the enhancement of the ion transfer process.^31^ The increased output signal from the Fe/Cu debris-laden and carbon black-laden oils is considered to be caused by a higher triboelectric generating capability of contaminant ingressions (Fe, Cu, and carbon particles) than of organic components (oil molecules),^52,53^ thereby enhancing contact electrification of O–S TENG. However, the output voltage signal then decreases at a higher fraction of contaminants, which is most likely due to the unavoidable adhesion of oil on the tube after the flow passes. For example, obvious contaminant adsorbates adsorbed on the inside walls of the nonmetal surfaces after testing paraffin oils with a high debris fraction (20 mg mL^–1^) as shown in Figure S12, so the contaminants with some charges remain on the surfaces, resulting in the partial screening of the tribo-charges on the films. Similarly, the orange color organic components in aged oils easily adsorb on the inner wall of O–S TENG after multiple-squeezing and -loosing processes such as the paraffin oil after the high aging time (192 h) (Figure S12).

**Figure 4:**
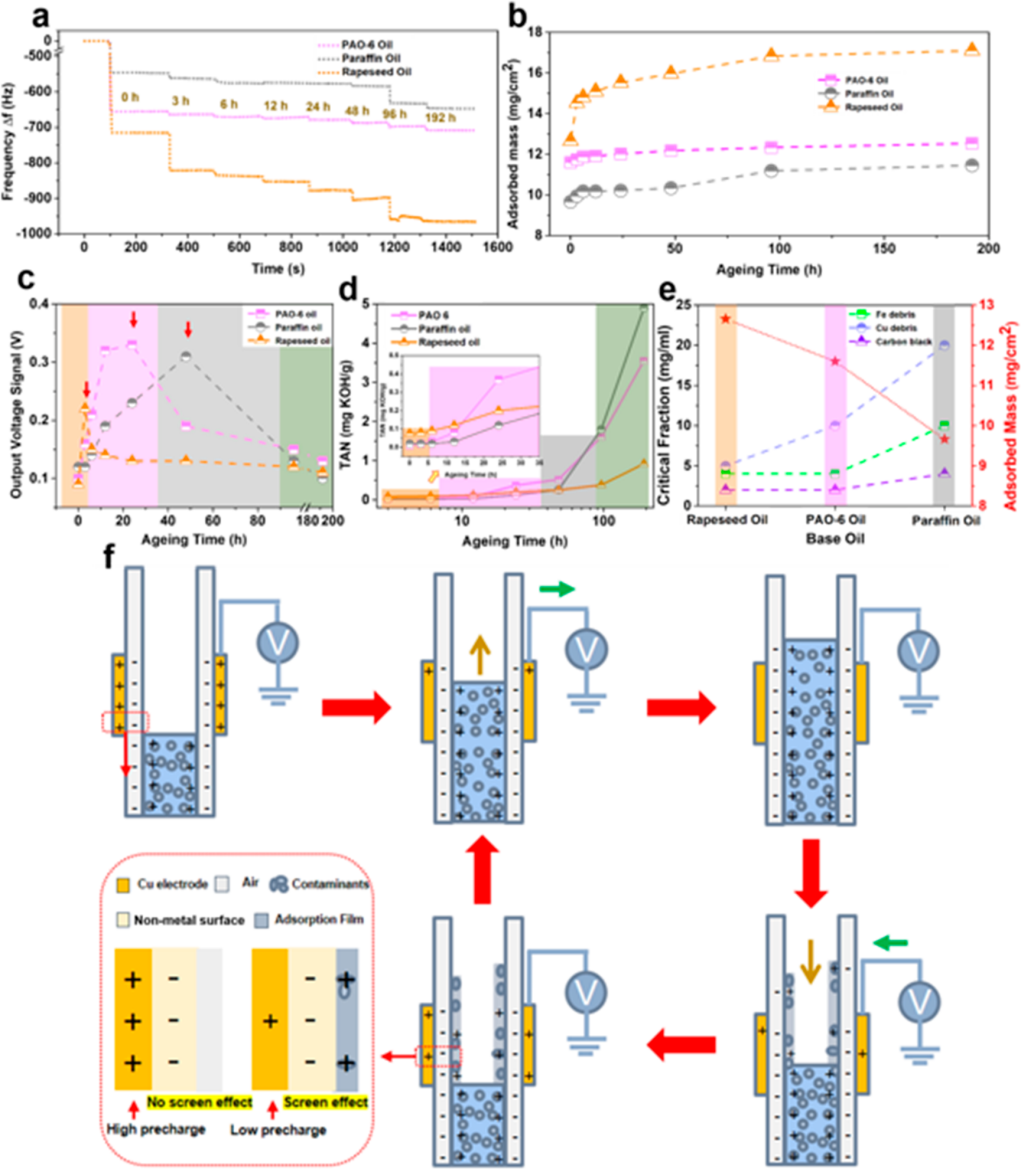
Adsorption behavior and electrical output mechanism of lubricating oils. (a) QCM-D data showing the change in normalized frequency with the increase in aging time. (b) Adsorption masses of oil molecules on Au substrate measured by QCM-D for base oils. (c) Output voltages of base oils as a function of aging time for PTFE-based O–S TENG. (d) TAN values of base oils as a function of aging time. (e) Critical fractions of incoming contaminants and adsorption masses of base oils. (f) Schematic diagram of the actual working principle of O–S TENG in contact with contaminant-laden oils and the charge distribution in different stages.

The adsorbing behavior of oil flows on surfaces play a critical role in the output capacity of TENG. Therefore, we quantitatively studied the adsorbing performance of lubricating oils. We use a quartz crystal microbalance with dissipation (QCM-D) to kinetically analyze the buildup of the adsorbed layers on the inner wall of O–S TENG.^54^ In Figure 4a, a rapid decrease in the frequency of pure base oils (0 h) is observed due to the adsorption of oil molecules to the substrate. The frequency values are very stable over time, and 300 s adsorption time is allowed to ensure the saturated oils adsorbed on the substrate. Then oils aged for a different period (3∼192 h) are investigated in sequence. For long aging times (96∼192 h), a significant decrease in the frequency is observed, which means a longer time-aging oil adsorbs more on the substrate. In addition, when base oils are injected into the QCM cell, the increase in dissipation caused by adsorption is obtained as shown in Figure S13. It can be clearly seen that the dissipation is small enough (Δ*D* < 10Δ*f*) (Δ*D*, dissipation; Δ*f*, resonance frequency), so the adsorbed film on the substrate is verified to be a rigid layer and the Sauerbrey equation can be used to calculate the adsorbed mass from the frequency change.^55,56^ The results of the adsorption mass are summarized in Figure 4b. It is found that the adsorption mass of these oils increases with the aging time. The aged paraffin oil has the lowest value of adsorption mass (11.5 mg cm^–2^) after a long aging time (192 h), and the adsorption mass of aged rapeseed oil is as large as 17.1 mg cm^–2^. During the whole aging time (0∼192 h), the adsorption mass of aged paraffin oil is the smallest. The aged rapeseed oil always has a much larger adsorption mass than the other two base oils, which further confirms the results in Figure 2 concerning the much lower output values of aged rapeseed oil than those both of aged paraffin oil and aged PAO-6 oil.

Although the output voltages of all aged base oils increase at first and then decrease (Figure 2), the critical aging time is different as shown in Figure 4c. The critical aging time of aged paraffin oil is as high as about 48 h, while the critical values of aged PAO-6 oil and rapeseed oil are 24 and 3 h, respectively. It is because aged paraffin oil is the most difficult to be adsorbed on the surface (Figure 4b), so that the screen effect is much weaker, and the output signal can maintain a high value despite the aging time increase. Comparatively, the corresponding adsorbed film of aged rapeseed oil with a strong adhesive effect can be easily deposited on the surfaces, so the screen effect is irreversible, and the output signal decreases quickly. Apart from the critical aging time, the output values at different aging period are also different for these base oils. It can be seen that the aged rapeseed oil has a little higher output value than both aged PAO-6 oil and aged paraffin oil during aging time from 0 to 5 h (Figure 4c). The total acid number (TAN) of aged rapeseed oil is much higher compared with that of the other base oils (Figure 4d) at the initial aging period, so the higher output capacity is attributed to the enhancement of ion transfer process for aged rapeseed oil in spite of the surface adsorption effect. With the aging time increasing (up to about 35 h), the TAN of aged PAO-6 oil sharply increases, meaning ion transfer plays a prominent effect for aged PAO-6 oil. For paraffin oil, when the aging time increases from 35 to 90 h, the TAN of aged paraffin oil is as large as that of aged PAO-6 oil, and also there is a much smaller value of adsorption mass for aged paraffin oil (Figure 4b); thereby, aged paraffin oil displays the highest output value. Furthermore, when the aging time is over the critical value (24 h for PAO-6 oil; 48 h for paraffin oil), the TAN increases sharply and the output voltages of aged PAO-6 oil and aged paraffin oil decrease quickly, which confirms that the increase of the aging degree leads to the excessive ions in the oil flows and will interfere with electron transfer process. For aged rapeseed oil, the TAN has no evident change with the increase of aging time, but the corresponding adsorption mass increases more obviously, which results in the decrease of the output signal due to the screen effect from the adsorption layers around O–S TENG.

The adsorption behaviors of lubricating oils not only play an important role in the output signal of aged oil, but also directly affect the output performance of Fe/Cu debris-laden and carbon black-laden oils. As shown in Figure 4e, the adsorption masses of pure rapeseed oil, pure PAO-6 oil, and pure paraffin oil are 12.7 mg cm^–2^, 11.6 mg cm^–2^, and 9.7 mg cm^–2^, respectively. Meantime, the critical fractions of contaminants (Fe debris, Cu debris, and carbon blacks) in turn increase for each base oil. The lubricating oil with larger adsorption mass can carry more contaminants to remain on the surface of O–S TENG, which will lead to the quick saturation of screen effect and to the decrease of output capacity. Therefore, although incoming contaminants can effectively contact the nonmetal dielectric surfaces and can provide a higher precharges of oil flows, the corresponding strong adsorbed oil film is easily deposited on the surfaces, so the screen effect is irreversible, and the output signal gradually decreases as shown in Figure 4f.

### Formulated Lubricating Oil Condition Monitoring by the O–S TENG

**Figure 5:**
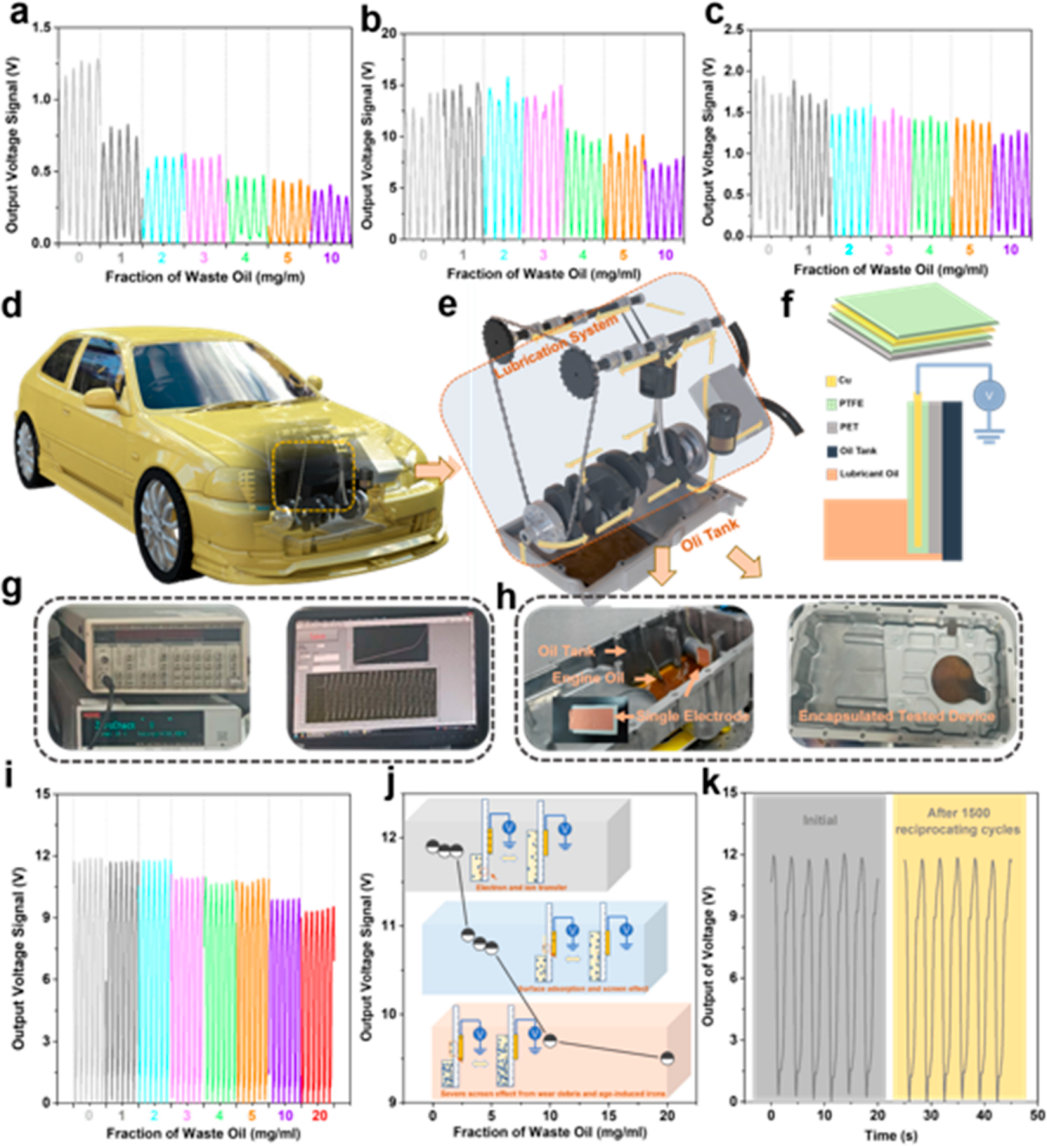
Output characterization of the O–S TENG monitoring formulated lubricating oils. The output voltage of a commercial engine lubricating oil in contact with PTFE-based (a), LDPE-based (b), and GLASS-based (c) O–S TENG as a function of the fraction of waste oil. A sketch of a vehicle corresponding to a lubrication system area (d). A sketch of oil circuit and oil tank in the lubrication system (e). A schematic diagram of a designed O–S TENG with a single electrode used in the industrial transportation system (f). Output monitor used for O–S TENG in this study (g). Actual tested devices of output signals for the transportation lubrication system, which includes engine lubricant oil, oil tank, and single electrode (h). Output voltage of the engine lubricating oil as a function of the fraction of waste oil (i,j). Output characteristic after stretching 1500 reciprocating times compared with initial output (k).

To monitor full formulated lubricating oils for industry application, a fresh formulated commercial engine lubricating oil with various fractions of waste oil (instead of pure base oils in the previous section) is used as a test object. As shown in Figure 5a–c, the output voltages of the oil are much higher than those of pure base oils shown in Figure 2 and Figure 3. Pure base oils are composed of nonpolar hydrocarbons and the signal output mainly results from a weak electron transfer process, so the output value is low (Figures 2 and 3). The commercial lubricating oils are composed of base oils and other components, such as active/polar additives, that is, antiwear, dispersing, and antirust additives,^57−59^ which can improve significantly the interfacial transfer of electron and ion. In addition, the charge trapping ability of the contact layer increases with the increased dielectric constant.^60^ The dielectric constant of the employed commercial engine oil is much higher than those of the base oils shown in Figure S14. Therefore, the output signals of commercial lubricating oils (more than 1.0 V) are much higher than those of pure base oils (about 0.1 V). The voltage values of engine lubricating oils finally decrease at a high fraction of waste oils, which are in good agreement with the output performance of contaminant-laden base oils. This occurs because more and more incoming contaminants such as wear debris are adsorbed on the dielectric surfaces with the increase of waste oil fraction, and also more age-induced ions with opposite charges are adsorbed onto the contacted surfaces, thereby screening the tribo-charges on the generation layer.

According to the above findings, it is believed that the designed O–S TENG could be applied in the actual field to realize the real-time and online detection system in industrial applications. To demonstrate this, a self-powered sensor is developed for real-time and online monitoring of the engine lubricating oil in an actual oil tank on a simulated test platform. The mechanical motion of vehicles always involves acceleration and deceleration on complex roads such as school roads, highways, and road intersections.^61^ Lubricating oils can generate an oil wave in the confined space of the vehicle tank because of the inertia. When the O–S TENG is attached to the inner wall of the tank, an output signal can be obtained from the contact-separation between the oil wave and the TENG solid surface. A vehicle sketch with the oil circuit and oil tank in the lubrication system is shown in Figure 5d,e. The real-time and online O–S TENG sensor comprises a short rectangular Cu electrode fully covered by a PTFE film that is attached to an inner wall of the tank (Figure 5f). The collected data is transferred to a computer to realize real-time display by the data capture device as displayed from the photography of device in Figure 5g. The tank shown in Figure 5h is driven by a linear motor to generate the oil wave (Supporting Movie 1, Movie 2, and Movie 3, and Movie 4). Figure 5i shows the output voltage of O–S TENG slightly decreases with a low fraction of waste oil (0∼2 mg mL^–1^), and significantly decreases with a fraction of waste oil from 3 mg mL^–1^ to 20 mg mL^–1^ (Movie 5, Movie 6, and Movie 7). This should be due to the screen effect of the incoming components, such as wear debris, deposited carbon, and age-induced oxygen-containing groups, adsorbed on the TENG surfaces (Figure 5j). When the temperature of oil increases, the output voltage decreases gradually (Figure S15). This is because the high temperature will cause thermionic emission which will lower the TENG output performance.^62^ The developed TENG is suitable to be used in the oil tank and will have little fire hazard because the output values of electric current and charge are only about ±0.4 nA and ±0.2 nC (Figure S16), which is safe for such kind of application. In addition, there is not a distinct change in the output values of the O–S TENG after the 1500 times cycles shown in Figure 5k, which means the transferred charges are also almost the same as that at the initial pristine state. Hence, reciprocating cycles have little effect on the adsorption behavior indicating the designed O–S TENG exhibits good ductility and stability.

## Conclusions

This study developed a self-powered triboelectric sensor for monitoring lubricating oils based on the oil–solid interfacing triboelectrification effect. The charge transfer between water and solids is suggested to have both electrons and ions exchange. But for the case of oil with solids, since there are no ions in oil, the surface charges on the solid after contacting the oil should be electron exchange from the oil molecules. Our experimental results carried out here suggest that electrons are transferred from oil to the glass surface, which is responsible for the signals we have detected. This finding is consistent with our previous study for the water–oil interface, in which electron transfer was also suggested.^63^ For the base oils (poly(α-olefin), paraffin, and rapeseed oils), the output voltage finally decreases at long aging time and high debris fraction due to the irreversible effect of screen film adsorbed on the electrode surface. We obtained the adsorption mass of these base oils on the substrate further confirming the screen effects by QCM-D analysis. On the basis of the findings, a self-powered monitor is successfully developed for real-time and online monitoring of the engine lubricating oil in an actual oil tank. It is believed that this self-powered triboelectric sensor has the great advantages of monitoring service performance of lubricating oils for different mechanical systems in a cost-efficient way.

## Methods

### Characterization

The output voltage signal was measured using a Keithley 6517 system electrometer. To measure the output performance of the O–S TENG in a transportation system, a linear motor from Wang’s group (LinMot, E1100, America) was used to drive the system to move periodically with the amplitude of 100 mm and cycle of 2.4 s, in which the position wait time of start or stop is 1 s. The micromorphology of this wear debris was taken by a scanning electron microscope (Quanta 200 FEG, FEI, America). Oxygen-containing functional groups of aged oils were investigated by a Fourier-transform infrared spectroscopy (Vertex, NETZSCH, Germany), and their total acid numbers (TAN) were obtained by a TAN tester (Delit, China). A QCM-D instrument (Q-sense E4 system, Biolin Scientific, Sweden) was used to simultaneously measure the changes of both resonance frequency (Δ*f*) and dissipation (Δ*D*) for the adsorption of oil components on a normative Au substrate. The dielectric constant of lubricating oil was measured by an automatic oil dielectric loss and volume resistivity tester (Delit, China)

### Experimental Section

Poly(α-olefin) and paraffin oils were purchased from Shanghai Qicheng Industrial Co., Ltd., P. R. China. Rapeseed oil was obtained from Arawan Co., Ltd., P.R. China. Before O–S TENG test, we kept these base oils at 105 °C for 5 h in a vacuum drying oven for dry processing. For dropper-based O–S TENG tests, the velocity and acceleration of the oil are about 3.0 cm/s and 6.0 cm^2^/s, relatively. The flow rate and volume of the oil in the dropper are about 0.6 mL/s and 2 mL at environment temperature (25 °C). Before QCM-D test, all these oils were fully diluted by petroleum (30 wt %) for achieving low viscous tested oils and stable adhesive data from QCM-D. The droppers and Cu electrodes were bought from Beijing Jiashitao Technology Co. Ltd., P.R. China. The full formulated commercial engine lubricant oil (0W-16) was achieved from Autobacs Quality Co. Ltd., P.R. China. The waste oil was acquired in a being-repaired motor vehicle from a dealership (Tianyuanbao Road Auto Maintenance Center, China).

### Fabrication of O–S TENG

There are two types of O–S TENG fabricated in this study. They are manual dropper-based and single electrode-based O–S TENGs. For the dropper-based O–S TENG, the width and area of the copper electrode covering the droppers are 15 mm and 263.8 mm^2^. The inner diameters of all droppers are 5 mm. The contact areas of the TENG in this study are all the same for avoiding their influence on the condition monitoring of oils. The Cu electrode with a width of 15 mm was uniformly attached on a dropper substrate. PTFE, LDPE, and GLASS are used as substrate materials as shown in Figure S1. The single electrode-based O–S TENG was first fabricated by preparing a long rectangular copper electrode with a width of 3 mm and a length of 5 mm. Each surface of the electrode was completely covered with an 80 μm thick PTFE film; meanwhile, the edges of the electrode were sealed by the PTFE film to prevent contact with oils, and one of the O–S TENG surfaces was attached to a double-sided foam tape substrate.

## Supporting Information Available

Table of factors of the contaminant ingressions studied for the different times in the atmospheric environment; additional figures supporting the text (PDF)
Front view of oil tank (MP4)
Nonencapsulated oil tank1 (MP4)
Nonencapsulated oil tank2 (MP4)
Encapsulated oil tank (MP4)
Data view1 (MP4)
Data view2 (MP4)
Data view3 (MP4)
