# Supplementary material for: Real-Time and Online Lubricating Oil Condition Monitoring Enabled by Triboelectric Nanogenerator
Source: ACS Nano. 2021 Jun 25;15(7):11869–79. doi: 10.1021/acsnano.1c02980 (PMC8320232; doi:10.1021/acsnano.1c02980)
Supplement: Supplementary file 1 — nn1c02980_si_001.pdf [file nn1c02980_si_001.pdf]

Supporting Information

Real-time and on-line lubricating oil condition monitoring enabled by triboelectric nanogenerator

Jun Zhao[a,b], Di Wang[a], Fan Zhang[c], Yuan Liu[d], Baodong Chen[d], Zhong Lin Wang[d,*], Jinshan Pan[e,*], Roland Larsson[a], Yijun Shi[a,*]

[a] Division of Machine Elements, Luleå University of Technology, Luleå, SE-971 87 Sweden
[b] College of Mechanical and Electrical Engineering, Beijing University of Chemical Technology, Beijing 100029, P. R. China
[c] Department of Engineering and Design, School of Engineering and Information, University of Sussex, Brighton, BN1 9RH, United Kingdom
[d] CAS Center for Excellence in Nanoscience, Beijing Key Laboratory of Micro-Nano Energy and Sensor, Beijing Institute of Nanoenergy and Nanosystems, Chinese Academy of Sciences, Beijing, 101400, P. R. China
[e] Division of Surface and Corrosion Science, Department of Chemistry, KTH Royal Institute of Technology, Stockholm, SE-100 44, Sweden

*Corresponding authors:
E-mail addresses: yijun.shi@ltu.se; jinshanp@kth.se; zlwang@gatech.edu

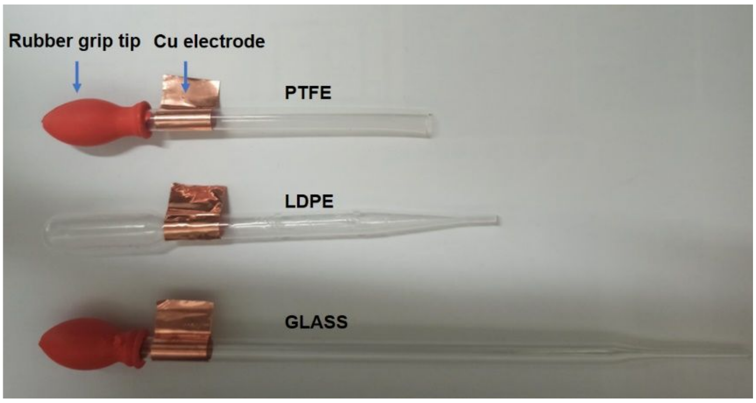

Figure S1. The photograph of the developed O-S TENG sensors fabricated with PTFE, LDPE and GLASS droppers as the substrates and Cu foil attached on the outer surface of the droppers.

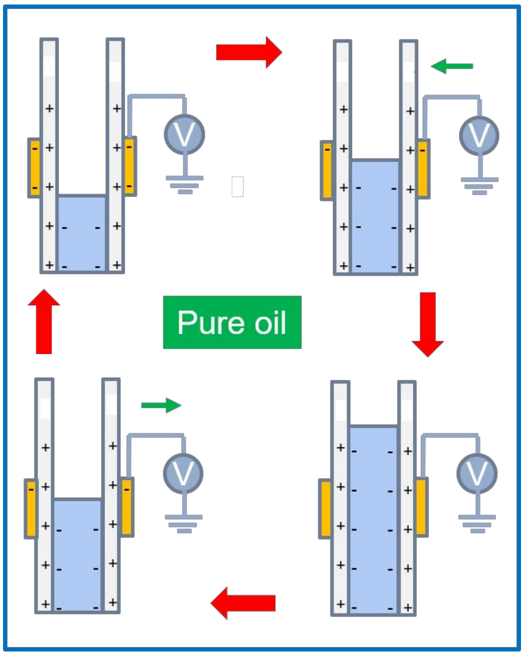

Figure S2 Structure illustration and working principle of the GLASS-based O-S TENG. Interacting with oil or copper, the GLASS surface retains a layer of positive charges. Electrons will flow from the Cu electrode to the ground under short-circuit condition with the oil flow contacting the non-metal layer. When the flow leaves the non-metal layer, electrons will flow from the ground to the Cu electrode to reach a new electric equilibrium.

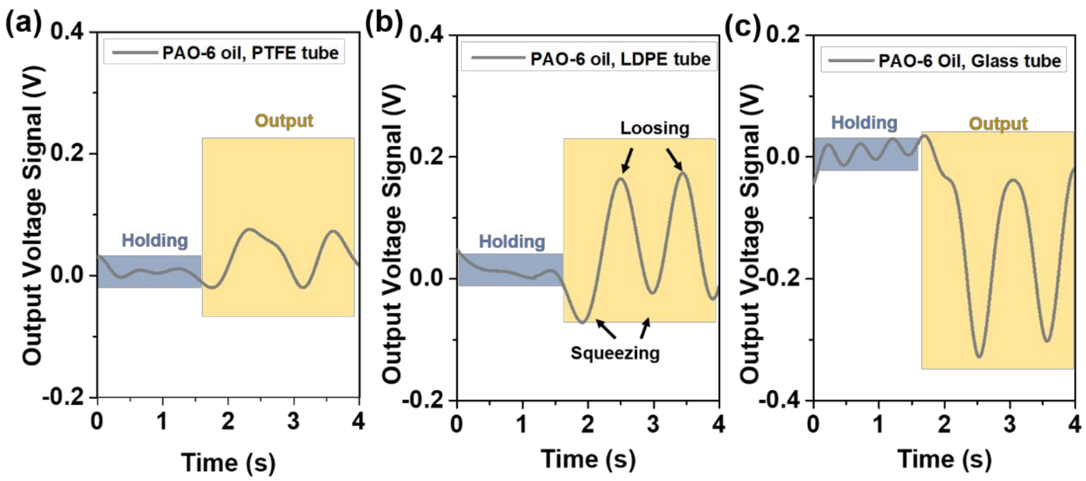

Figure S3 An initial output of open-circuit voltage at the very beginning cycles of squeezing and loosing after holding stage.

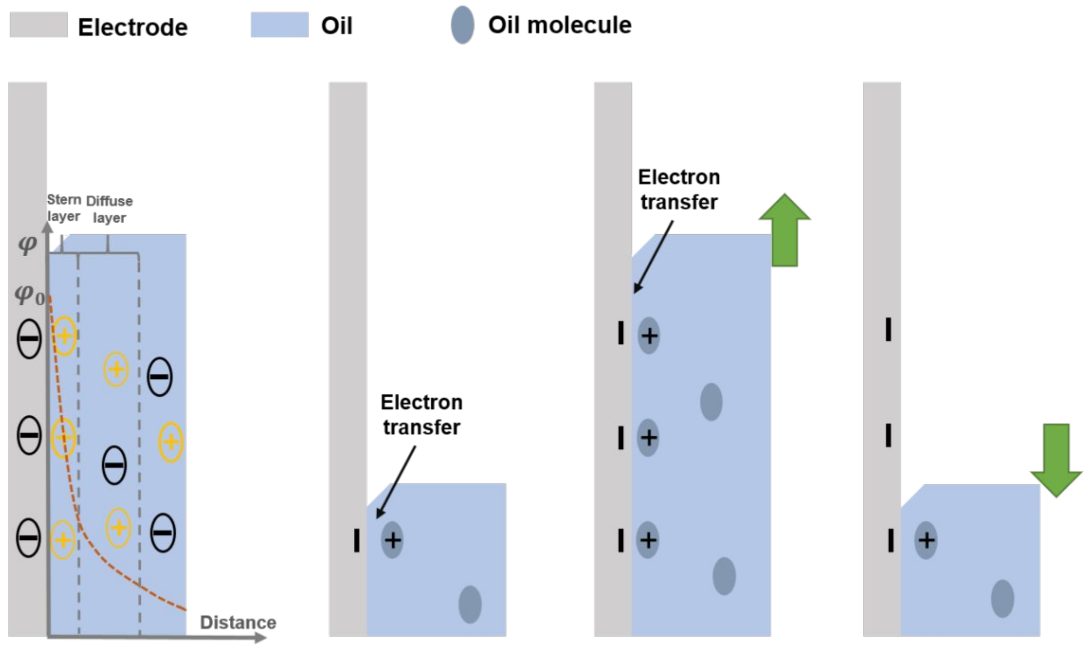

Figure S4 Schematic representation of the initial electron transfer for the contact electrification between the oil and the surface (for PTFE and LDPE)

Table S1. The factors of the contaminant ingressions studied for the different time in the atmospheric environment. Iron and copper particles used to simulate the wear debris, carbon black to simulate the carbon deposition. Heating was applied to accelerate oil ageing.

	PAO 6		Paraffin oil			Rapeseed oil	
Aging under 150 ℃ (h)	3,	6,	12,	24,	48,	96,	192
Iron particles (mg/ml)	1,	2,	3,	4,	5,	10,	20
Copper particles (mg/ml)	1,	2,	3,	4,	5,	10,	20
Carbon black (mg/ml)	1,	2,	3,	4,	5,	10,	20
Fuel oil (Diesel, wt%)	5		10		20		30
Moisture (Water, wt%)	0.01, 0.025, 0.05, 0.10, 0.25, 0.50, 1.00						

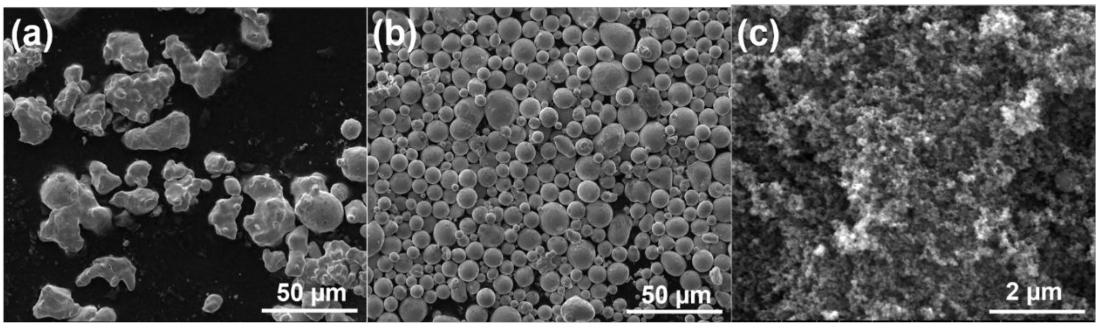

Figure S5. SEM morphologies of Fe particles (a), Cu particles (b) and carbon blacks (c) used in this work.

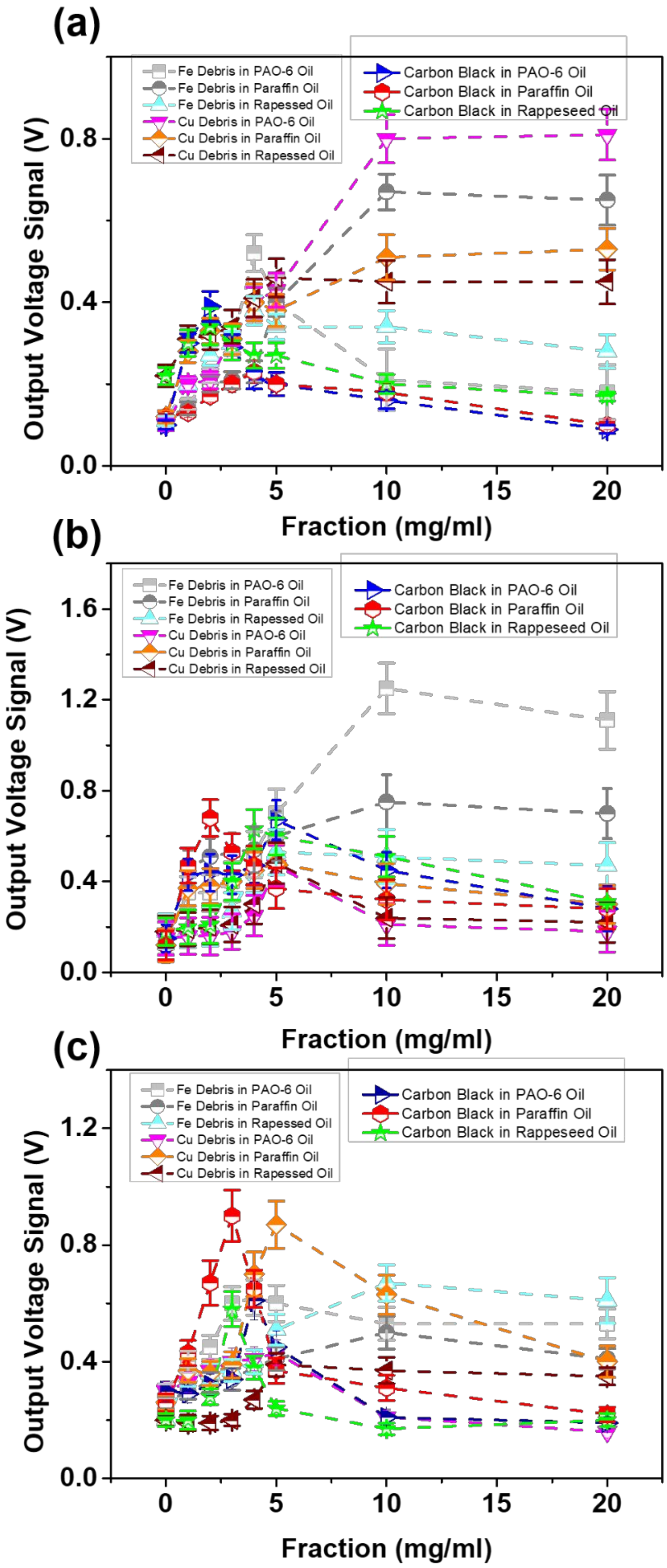

Figure S6. The output voltage of PTFE-based (a), LDPE-based (b) and GLASS-based (c) O-S TENGs as a function of the contaminant fraction (Fe debris, Cu debris and carbon blacks) in base oils.

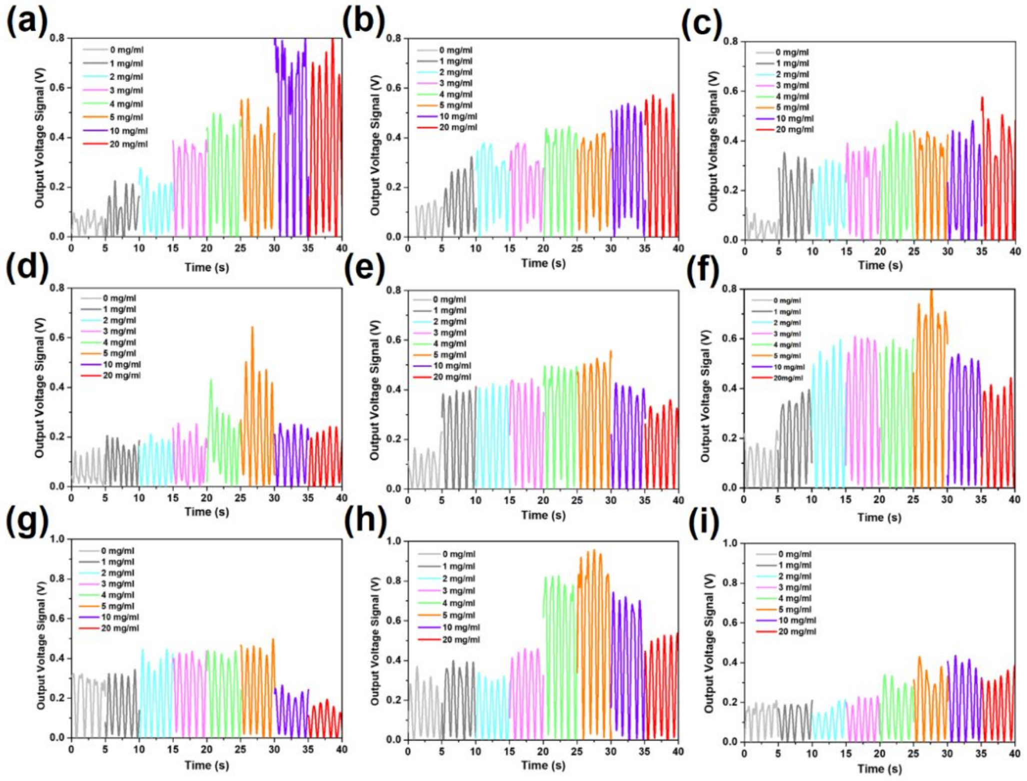

Figure S7. Various fraction of Cu debris (0~20 mg/ml) affecting on the output voltage of PTFE-based O-S TENG monitoring PAO-6 oil (a), paraffin oil (b) and rapeseed oil (c). The fraction affecting on the output of LDPE-based O-S TENG monitoring PAO-6 oil (d), paraffin oil (e) and rapeseed oil (f). The fraction affecting on the output of GLASS-based O-S TENG monitoring PAO-6 oil (g), paraffin oil (h) and rapeseed oil (i).

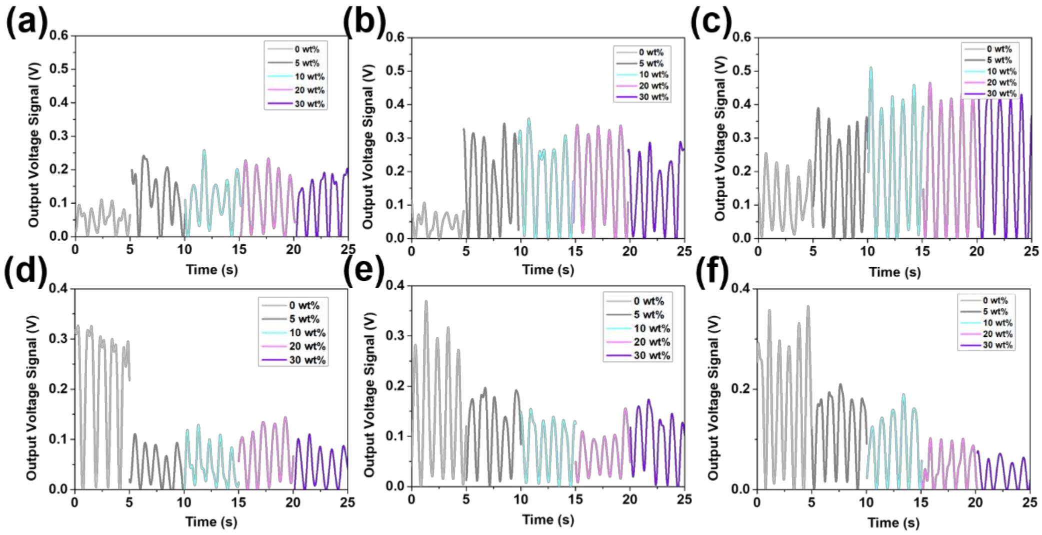

Figure S8. Various fraction of diesel oil (0~30 wt%) affecting on the output voltage of LDPE-based O-S TENG monitoring PAO-6 oil (a), paraffin oil (b) and rapeseed oil (c). The fraction of diesel oil affecting on the output of GLASS-based O-S TENG monitoring PAO-6 oil (d), paraffin oil (e) and

rapeseed oil (f).

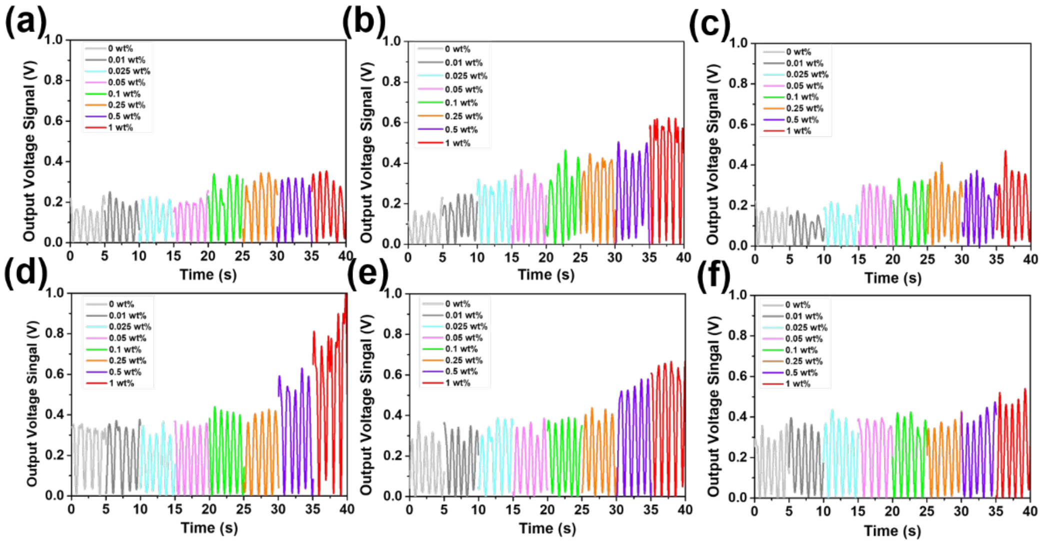

Figure S9. Various fraction of water (0~1 wt%) affecting on the output voltage of LDPE-based O-S TENG monitoring PAO-6 oil (a), paraffin oil (b) and rapeseed oil (c). The fraction affecting on the output of GLASS-based O-S TENG monitoring PAO-6 oil (d), paraffin oil (e) and rapeseed oil (f).

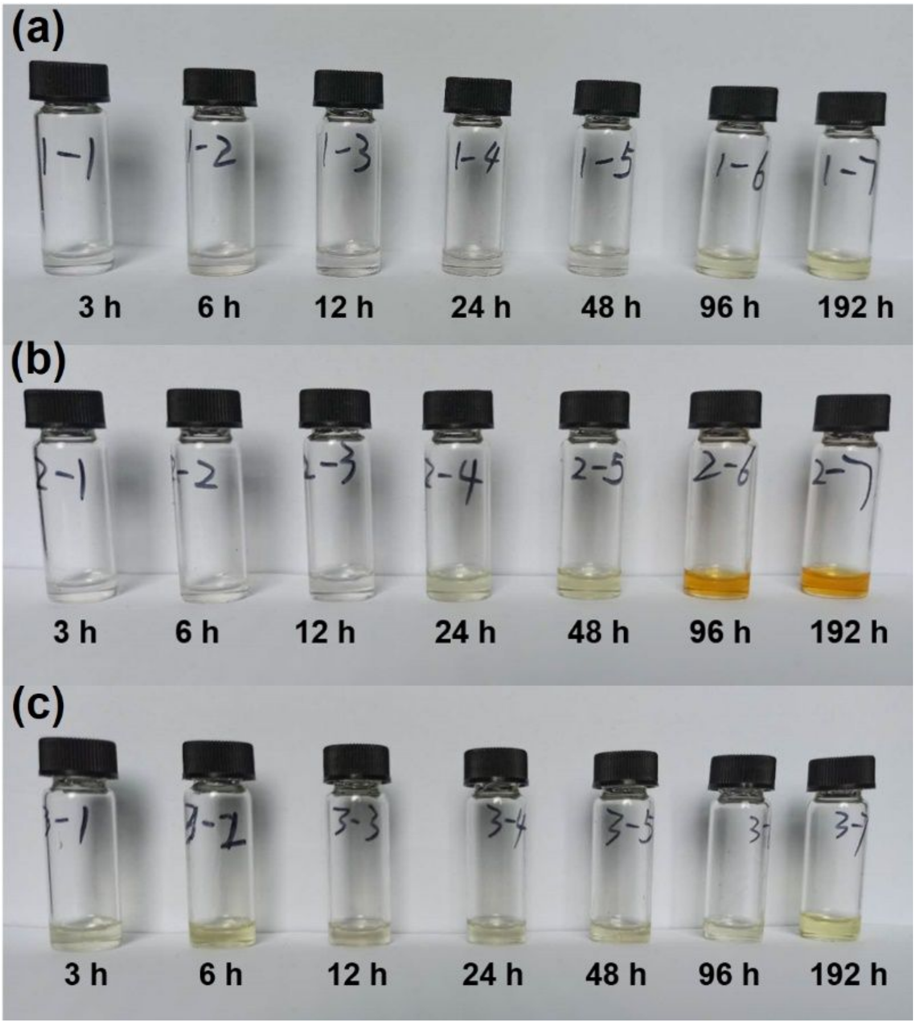

Figure S10. Optical microscope photographs of PAO-6 (a), paraffin (b) and rapeseed (c) oils aged at different time

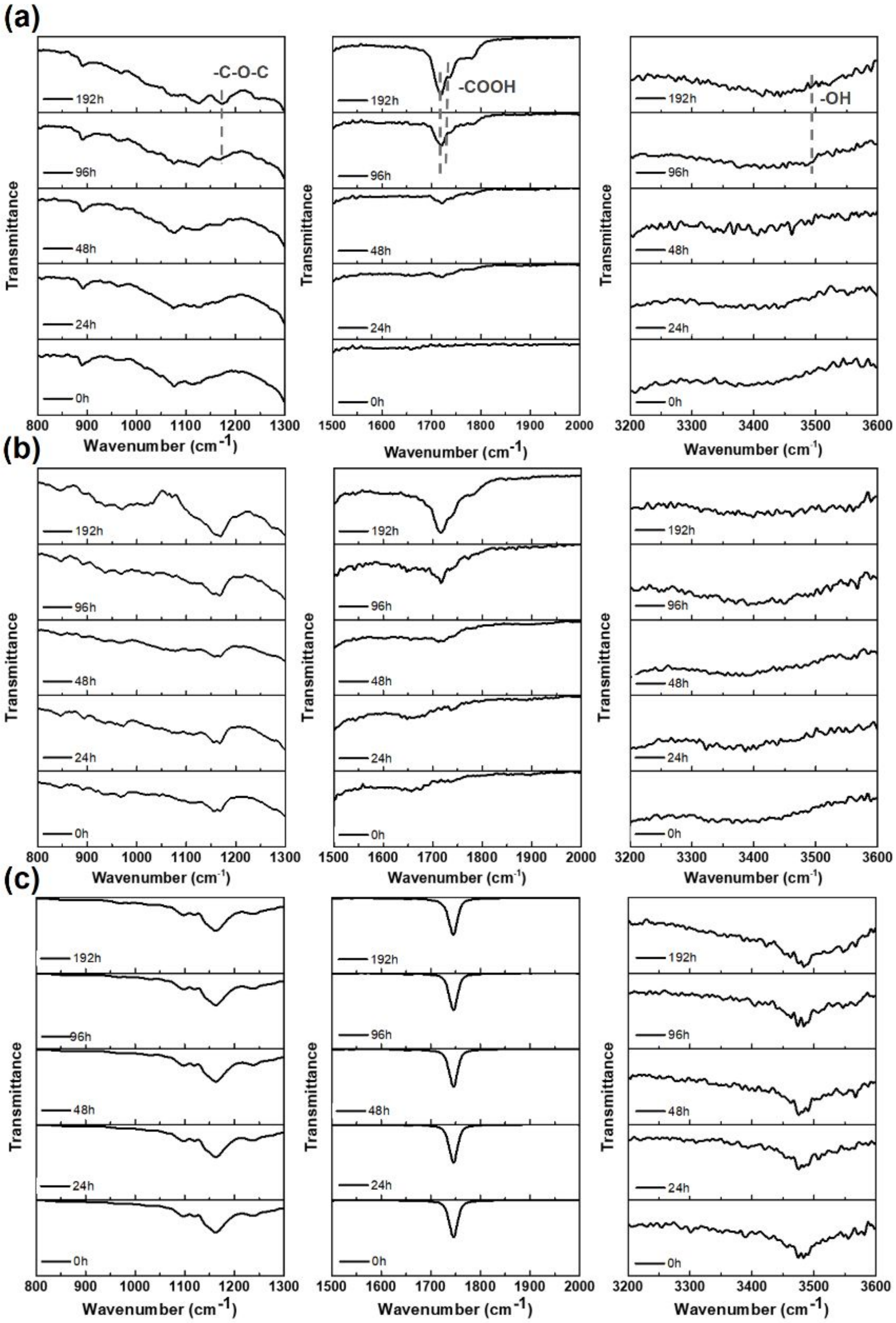

Figure S11. FTIR spectrum of base oils (PAO-6 (a), paraffin (b) and rapeseed (c) oils) at different ageing time

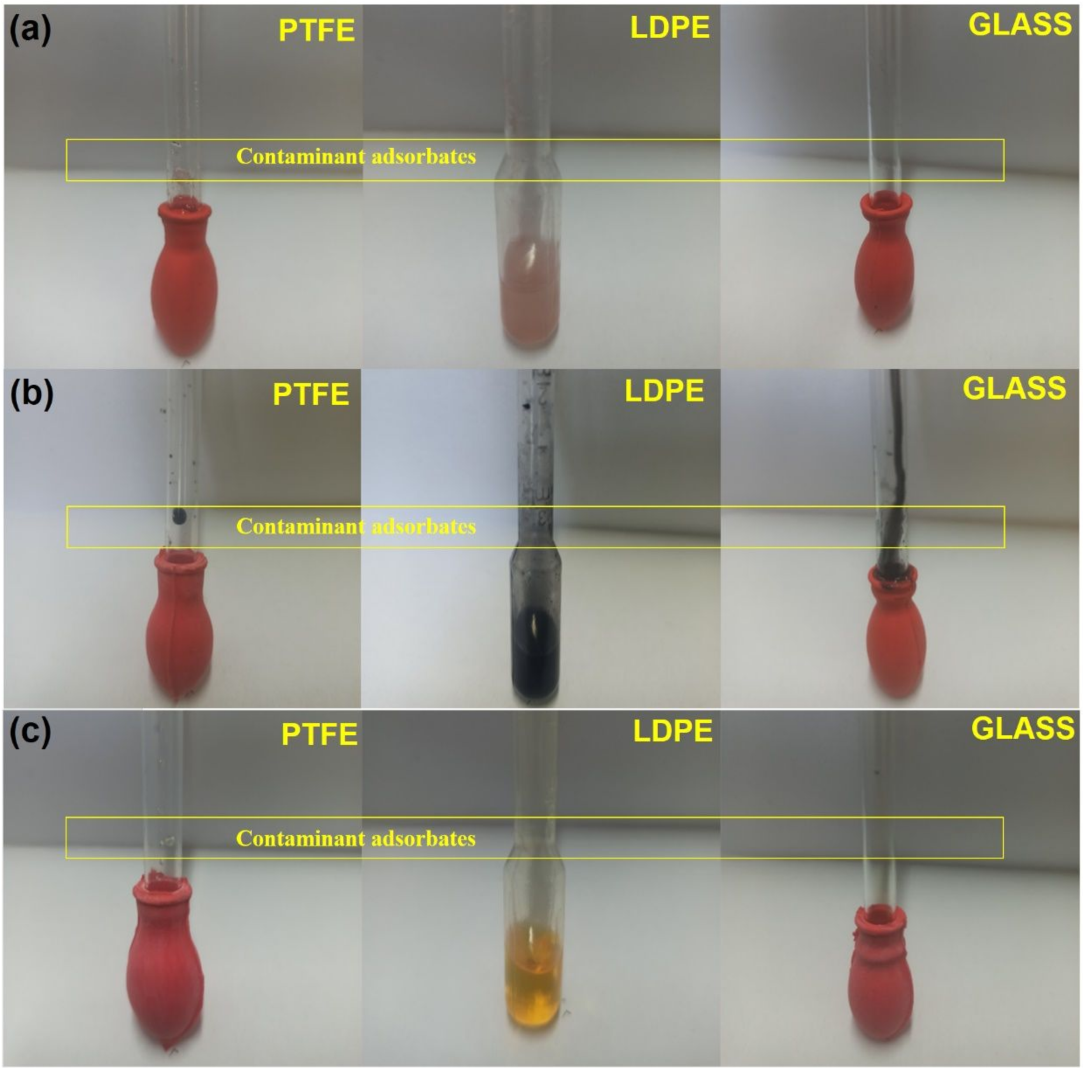

Figure S12. Optical microscope photographs of O-S TENG after tests: PTFE-based, LDPE-based and GLASS-based O-S TENGs of paraffin oil with Fe debris (20 mg/ml) (a); PTFE-based, LDPE-based and GLASS-based O-S TENGs of paraffin oil with carbon blacks (20 mg/ml) (b); PTFE-based, LDPE-based and GLASS-based O-S TENGs of aged paraffin oil at 150℃ for 196 h (c).

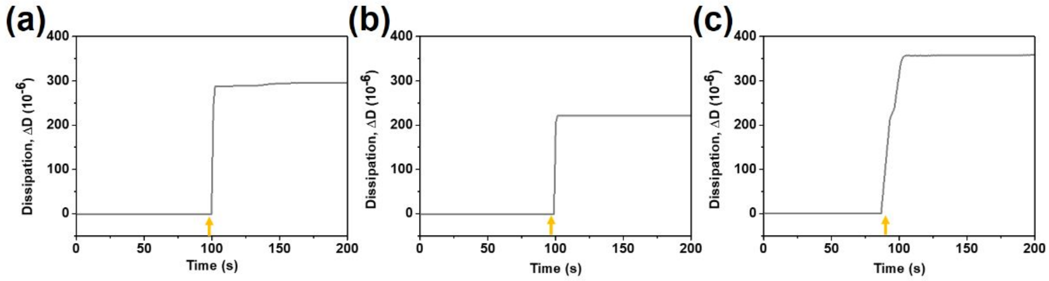

Figure S13. Change in dissipation (ΔD) of the substrate surface upon adsorption of PAO-6 (a), paraffin (b) and rapeseed (c) oils. The arrow indicates the time that the oil was injected into the cell.

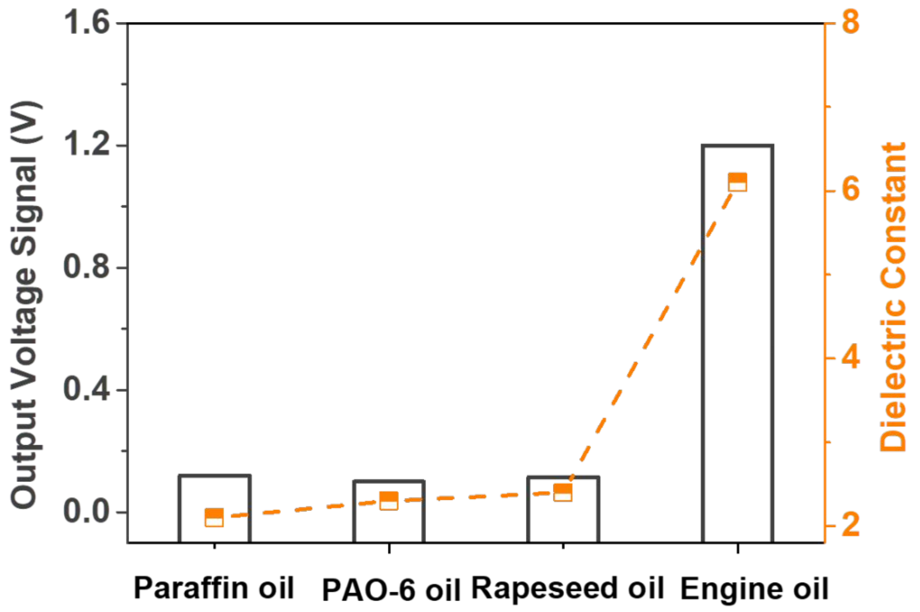

Figure S14 The dielectric constants and output voltages of base oils and engine oil used in this study

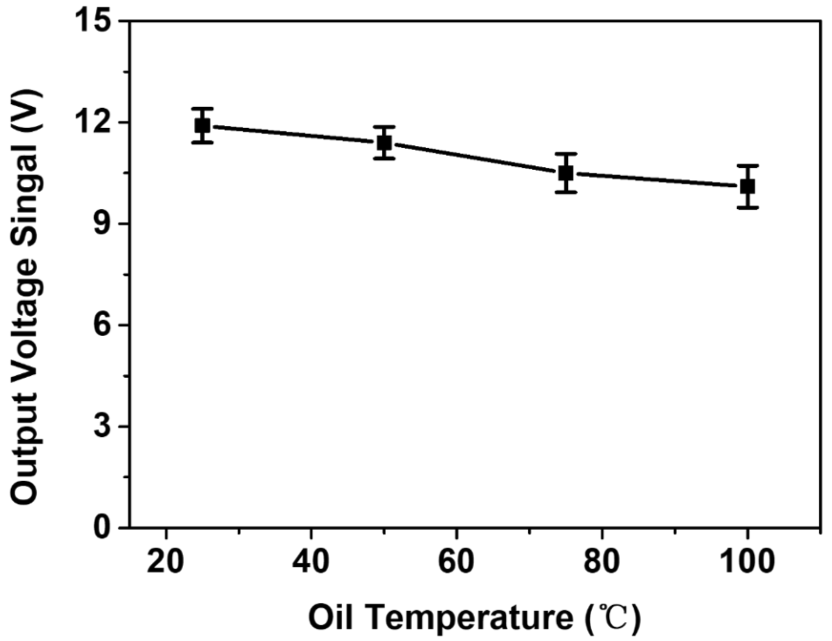

Figure S15 Output voltage of the engine lubricating oil as a function of oil temperature

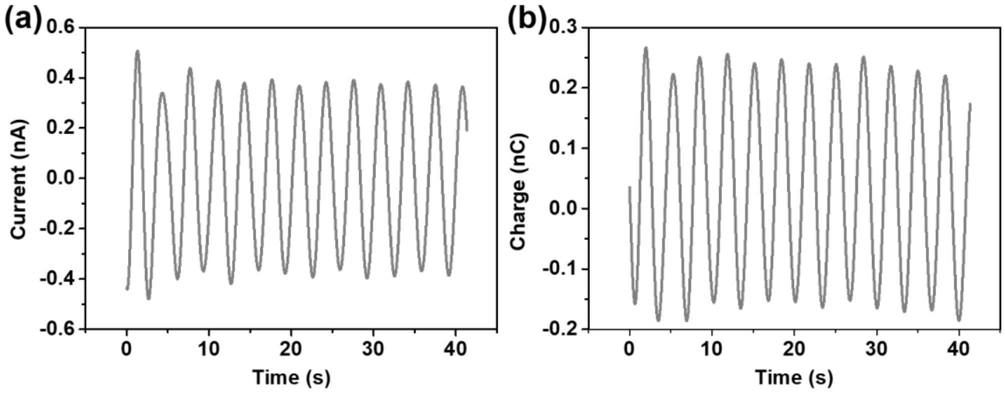

Figure S16 Short-circuit current (a) and charge generation (b) of the engine lubricating oil in the oil tank.
